# Narrowly and Broadly Tuned Peripheral Gustatory Neurons Cooperate to Encode Three Behaviorally Relevant Stimulus Categories

**DOI:** 10.1523/ENEURO.0306-25.2026

**Published:** 2026-08-19

**Authors:** Tao Huang, Robin F. Krimm

**Affiliations:** Department of Anatomical Sciences and Neurobiology, University of Louisville School of Medicine, Louisville, Kentucky 40202

**Keywords:** afferent, geniculate neuron, morphology, taste, tongue

## Abstract

Peripheral taste neurons vary in tuning breadth; some respond selectively to a single stimulus (narrowly tuned), and others respond to multiple stimuli (broadly tuned). How these differences contribute to taste coding remains unclear. We recorded calcium responses from 280 geniculate ganglion neurons to five or seven taste stimuli in male and female mice. Using five stimuli, 47% of neurons were narrowly tuned, while 53% responded to multiple stimuli. Hierarchical cluster analysis revealed five functional groups with five stimuli and six with seven stimuli, suggesting that the number of clusters is sensitive to the stimulus set rather than representing a fixed number of biologically distinct taste neuron “types.” Principal component analysis revealed three stimulus categories: (1) bitter, sour, and nonsodium salts (aversive), (2) sucrose and umami (caloric/appetitive), and (3) sodium salts (sodium-specific). Both narrowly tuned and broadly tuned neurons within a single category contributed to this categorization. In contrast, 40% of the neurons were broadly tuned across categories and did not cluster with specific stimulus types. These cross-category neurons were more likely to exhibit mixture subadditivity—lower than additive responses—suggesting they could have a distinct role in encoding intensity or chemical context. Finally, we show Type II taste bud cells are required for representing these categories and mediating mixture subadditivity between sweet and sour stimuli. These results suggest peripheral gustatory neurons primarily function to categorize taste stimuli into three groups. Furthermore, differing roles for narrowly tuned and broadly tuned neurons may be a key organizing principle of peripheral taste representation.

## Significance Statement

How the peripheral gustatory system encodes taste information remains unclear. We found that both narrowly tuned and broadly tuned peripheral taste neurons categorize seven taste stimuli into three groups: innately aversive, innately appetitive, and sodium specific. These three categories are behaviorally relevant but are not single taste qualities. However, some broadly tuned neurons respond to stimuli across categories and have a subadditive response to mixtures, suggesting a separate role for these neurons, perhaps representing stimulus context. Finally, mixture subadditivity for some stimuli requires the convergence of sensory information from Type II and Type III cells. These findings are contrary to traditional models focusing on taste quality and instead support a focus on hedonics and contextual representations within peripheral taste neurons.

## Introduction

Peripheral gustatory neurons respond to a wide array of chemical stimuli, which are transduced by cells within the taste bud, conveying taste information to the brain. A central question in taste research is how these neurons represent taste quality. Numerous studies have shown that peripheral gustatory neurons vary in their tuning properties: while some neurons respond selectively to a single stimulus (narrowly tuned), others respond to multiple taste stimuli spanning different qualities (broadly tuned; Pfaffmann, 1941; Frank, 2000; Yoshida et al., 2006; Yoshida and Ninomiya, 2010; Barretto et al., 2015; Wu et al., 2015). This heterogeneity has been used to support differing coding strategies for early taste processing (Erickson, 1968; Erickson et al., 1980; Contreras and Lundy, 2000; Spector and Travers, 2005; Yarmolinsky et al., 2009).

The mechanisms by which peripheral taste neurons encode taste qualities have long been debated. The across-fiber model suggests that taste qualities are represented by patterns of activity across populations of neurons, each responding to multiple taste stimuli to varying degrees (Erickson, 1968; Erickson et al., 1980). In contrast, the labeled-line model proposes that individual taste qualities (sweet, sour, salty, bitter, umami) are conveyed by distinct, selectively tuned neurons (Yarmolinsky et al., 2009). While most researchers agree that the system likely contains elements of both strategies (Ohla et al., 2019), the idea that neurons can be divided into groups corresponding to basic taste qualities has strongly influenced the field. For example, studies have attempted to classify taste neurons in the geniculate ganglion based on gene expression profiles (Dvoryanchikov et al., 2017; Zhang et al., 2019; Anderson and Larson, 2020), with some suggesting that peripheral gustatory neurons fall into five basic taste-related categories, plus an additional group responsive to touch (Zhang et al., 2019; Anderson and Larson, 2020). However, this focus on five categories often overlooks the broadly tuned neuron population and likely underestimates the complexity of peripheral taste coding. Both the labeled-line and across-fiber viewpoints essentially rely on patterns of neural activity to represent stimulus identity (Spector and Travers, 2005) and assume that encoding taste quality (e.g., sweet, sour, salty) is the primary function of peripheral taste neurons.

However, peripheral taste neurons contribute to more than just the encoding of basic taste qualities. One example is their role in mixture subadditivity—a phenomenon in which the response of a neuron to a mixture of taste stimuli is smaller than the sum of its responses to individual components. Mixture subadditivity has been observed in electrophysiological recordings from taste bud cells and nerve fibers (Formaker and Frank, 1996; Frank et al., 2003; Talavera et al., 2008; Formaker et al., 2009; Breza and Contreras, 2012) and allows mixtures to be represented in a way that does not simply reflect the sum of their individual components. This phenomenon may sharpen sensory discrimination in the context of complex, multitaste stimuli.

Despite the complexity of taste circuitry and function, prior studies have primarily focused on narrowly tuned neurons, often treating them as the principal carriers of taste quality information (Yarmolinsky et al., 2009; Barretto et al., 2015). Broadly tuned neurons have historically been underemphasized or dismissed as noisy or less informative, based on the assumption that specificity is required for reliable taste discrimination (Spector and Travers, 2005). While these assumptions are not made in pattern coding, the emphasis is still on quality coding (Ganchrow and Erickson, 1970; Wu et al., 2015), rather than considering other functional roles.

Our goal was to reexamine the issue of peripheral taste representation. Similar to hamsters (Frank et al., 1988), we found in mice that rather than representing basic tastes, peripheral neural responses primarily divide stimuli into three categories: innately appetitive, innately aversive, and sodium specific. These categories suggest that the primary function of the peripheral taste system may be to encode the behavioral significance of stimuli rather than taste quality as defined by human psychophysics. Furthermore, many neurons (40%) are broadly tuned and respond to stimuli from multiple categories. We propose that these neurons contribute to taste coding in ways other than stimulus identification, such as mixture subadditivity, intensity, and the contextual modulation of sensory experience.

## Materials and Methods

### Animals

For mice used in calcium imaging experiments, Phox2b-Cre mice (Mutant Mouse Resource and Research Centers; MMRRC strain 034613-UCD, NP91Gsat/Mmcd, RRID: MMRRC_034613-UCD) were bred with Cre-dependent GCaMP6s mice [Ai96(RCL-GCaMP6s), Jax #028866]. *Phox2b-Cre* mice were genotyped using universal Cre primers (Cre1: GAGTGATGAGGTTCGCAAGA; Cre2: CTACACCAGAGACGGAAATC). *GCaMP6s* mice were genotyped according to the Jackson Laboratory protocol for strain 028866. These mice were referred to as Phox2b-GCaMP6s (*n* = 14).

For experiments using *Pou2f3*^−/−^ mice, we obtained mice from Ichiro Matsumoto at the Monell Chemical Senses Center (Matsumoto et al., 2011); *Pou2f3*^−/−^ mice were genotyped using the following primers: SK1 (int6-s), 5′-CATGCTGAGATTTGTCCCAAGCTG-3′; SK2 (int7-as), 5′-ATCTGCGCACAGCCTTGTATCTTC-3′; SK3 (neo-s2), 5′-ACAAGATGGATTGCACGCAGGTTC-3′; and SK4 (neo-as2), 5′-GTCCAGATCATCCTGATCGACAAG-3′. To generate experimental animals, homozygous *Pou2f3*^−/−^ mice were bred with Phox2b-Cre mice and homozygous GCaMP6s mice, producing offspring that carried either Cre or GCaMP6s and were all heterozygous for *Pou2f3*^+/−^. In *Pou2f3*^+/−^:GCaMP6s mice, the primers we used could not distinguish heterozygous from wild-type animals because the GCaMP6s allele also contains a neomycin resistance cassette. To avoid the need to distinguish these genotypes within the same litter, both *Pou2f3*^+/−^:GCaMP6s and Phox2b-Cre:*Pou2f3^+/−^* mice were bred with *Pou2f3*^−/−^ mice which generated litters in which all pups were either heterozygous or homozygous knock-out for *Pou2f3*. These genotypes were distinguished by the presence or absence of the wild-type *Pou2f3* allele. *Phox2b-Cre*:*Pou2f3*^−/−^ mice were bred with *Pou2f3*^−/−^:*GCaMP6s* mice. All resulting offspring were genotyped for the presence of *Cre*, *GCaMP6s*, and absence of wild-type *Pou2f3*. Because of this breeding strategy, these animals could not be compared with littermate controls.

Both male and female mice were used, and no systematic differences were observed. Animals were cared for and used in accordance with the guidelines of the US Public Health Service Policy on Humane Care and Use of Laboratory Animals and the NIH Guide for the Care and Use of Laboratory Animals.

### Immunohistochemistry and confocal imaging

*Pou2f3*^−/−^ and wild-type mice were killed with isoflurane followed by transcardial perfusion with 4% PFA. Tongues were dissected, and a razor blade was used to remove the tongue tissue caudal to and including the circumvallate papilla. The tongues were then carefully split down the midline with a razor blade under a dissection microscope. Dissected tissues were postfixed in 4% PFA overnight and transferred to 30% sucrose at 4°C overnight. Tongues were then frozen in optimal cutting temperature (OCT) compound and stored at −80°C before sectioning on a cryostat. Half tongues were sectioned at 70 µm sagittally from the midline to the lateral edge. Sections were washed for 15 min (two times) in 0.1 M PB. Tissues were incubated in blocking solution (3% donkey serum, 0.5% Triton X-100 in 0.1 M PB) at 4°C overnight and incubated for 5 d at 4°C with primary antibodies phospholipase Cβ-2 (PLCβ2; 1:500, RRID:AB_2630573; Santa Cruz Biotechnology), carbonic anhydrase 4 (Car4; 1:500, RRID:AB_10013483; R&D Systems), and Troma-1 (K8; 1:50, RRID: AB_531826; Developmental Studies Hybridoma Bank) in antibody solution (0.5% Triton X-100 in 0.1 M PB). Tissues were rinsed four times for 15 min each with 0.1 M PB, incubated with secondary antibodies (1:200, Alexa Fluor 488, Invitrogen A21208; 1:200, Alexa Fluor 555, Invitrogen A31572; and 1:200, Alexa Fluor 647, Invitrogen A21447). Sections were rinsed again (four times for 15 min each with 0.1 M PB), then mounted with Fluoromount-G, and coverslipped (high precision, 0107242; Marienfeld).

Taste bud images were obtained using an Olympus FluoView FV3000 confocal laser-scanning microscope with a 60× NA 1.5 lens (taste buds), using a zoom of 3, Kalman 2. Image sizes were initially set at 1,024 × 1,024 pixels. All colors were imaged sequentially in separate channels to avoid bleed-through.

### Calcium imaging

Mice were sedated with a 0.32 mg/kg injection of Domitor (medetomidine hydrochloride) intramuscularly and anesthetized with 40 mg/kg Ketaset (ketamine hydrochloride). Heart rate and blood oxygen saturation were monitored continuously with a pulse oximeter. Puralube was applied to the eyes to prevent drying while maintaining optical clarity. A rectal temperature probe was gently inserted to monitor and control the mouse's core temperature at ∼35–36°C, via a heating pad that was automatically adjusted based on the animal's core temperature throughout surgical procedures and recording sessions. The mouse's trachea was cannulated to expedite oral stimulation during recording and/or to deliver isoflurane. The esophagus was exposed with blunt dissection, and a small-diameter cannula was inserted into a slit in the esophagus and fed forward to the oral cavity. This cannula served as the delivery system for orosensory stimuli as reported previously (Wu et al., 2015). Once the tracheal and esophageal tubes were placed, the animal was flipped over to surgically access the geniculate ganglion. At that point, anesthesia was switched to isoflurane inhalation (1–3%) for maintenance, delivered by a vaporizer.

The geniculate ganglion was exposed using a dorsolateral approach. Briefly, with the skull firmly attached to a stereotactic head holder bar, the musculature overlying the zygomatic arch and the coronoid process of the mandible was removed with a cautery tool. We then drilled and removed the zygomatic arch and the coronoid process to expose the cranium. After cleaning the cranial surface of muscle and connective tissue, we carefully cut a rectangular window with a microdrill, removed the cranial piece, and aspirated a portion of the cortex overlying the geniculate ganglion. This approach has been used previously for the trigeminal ganglion (Leijon et al., 2019). By tilting the head with the nose elevated relative to the back of the head, the geniculate ganglion and the greater superficial petrosal (GSP) nerve were exposed. Once exposed, the preparation was transferred to our custom epifluorescence microscope and positioned under a 10× Super Apochromatic Microscope Objective, 0.50 NA, 7.77 mm WD (TL10X-2P, Thorlabs). The cell bodies of peripheral taste neurons in the geniculate ganglion were imaged for Ca^2+^ responses during automated stimulation of the tongue. Taste stimuli were delivered using a ValveBank programmable perfusion system with eight channels (AutoMate Scientific), one of which held artificial saliva and the others held taste stimuli. All solutions were mixed and delivered at room temperature of 26°C. Artificial saliva in one reservoir was perfused constantly over the tongue surface. For 10 s every minute, the solenoid valve for artificial saliva closes and one for a taste stimulus opens. Once this occurs, a taste stimulus was infused over the tongue through the same infusion pump, such that the flow rate is held constant. Because it is programmable once stimulation begins, it continues until the end of the experiment, so there is no variation in the time between stimuli for all three presentations. The stimuli, selected based on EC_50_ concentrations described by Wu et al. (2015), included sodium salts (142 mM NaCl and 142 mM Na acetate or NaCl with 5 µM benzamil), sour (10 mM citric acid), bitter mix (0.2 mM quinine and 1.7 µM cycloheximide), sweet (137 mM sucrose), umami (61 mM monosodium glutamate + 1 mM IMP or 61 mM monosodium glutamate + 1 mM IMP with 5 µM benzamil), and nonsodium salt (142 mM NH_4_Cl). Because EC_50_ values for Na acetate and ammonium chloride were not included in Wu et al. (2015), we used equal molar concentrations (142 mM) for NaCl, NH_4_Cl, and Na acetate. Equal molar concentrations are often used to compare salts (Formaker and Hill, 1988; Kloub et al., 1997; Yokota and Bradley, 2016). Mixtures were set at the same concentrations as the individual stimuli so that mixture subadditivity could be examined.

Video recordings were performed during all trials using a scientific complementary metal-oxide-semiconductor video camera (pco.panda cs4, excelitas, Pittsburgh) at 2 fps. This system is similar to those used in other published imaging studies of the geniculate ganglion (Lee et al., 2017; Zhang et al., 2019). The camera recorded all responses for the duration of the experiment.

### Experimental design and statistical analysis

#### Calcium image analysis

Image stacks were corrected for movement artifacts using the TurboReg image plugin in ImageJ (Wu et al., 2015; Leijon et al., 2019). ROIs were selected in ImageJ using the magic wand tool (Wu et al., 2015; Ghitani et al., 2017; Leijon et al., 2019). Custom MATLAB scripts (MathWorks), made publicly available (https://github.com/Dalston817/calciumImagingROITools), were used to calculate the change in fluorescence (Δ*F*/*F*0; Wu et al., 2015; Leijon et al., 2019). The fluorescence signal of a C-shaped area surrounding each ROI was subtracted from the cell response to remove potential contributions of out-of-focus signals (Yarmolinsky et al., 2016; Ghitani et al., 2017). Peripheral taste neurons were considered responsive to a given stimulus if Δ*F*/*F*0 was greater than five times the standard deviation (Leijon et al., 2019) of the 20 s baseline preceding the stimulus. Two trials for each stimulus were averaged (data for peak Δ*F*/*F*0 can be found at https://data.mendeley.com/datasets/nry4p7yy8k/1), and only ROIs where both responses were significantly above baseline were considered significant.

ROIs with no significant responses were not analyzed further and are not reported. ROIs from 150 neurons in four mice (two male, two female) responding to seven taste stimuli (including NH_4_Cl and NaAc) and ROIs from 135 neurons from five mice (two male, three female) responding to five taste stimuli plus two mixtures were analyzed. Because five of these stimuli were the same in these two groups, they were combined for analysis of five stimuli. Of the 285 ROIs collected, 280 responded significantly to at least one of the following five stimuli: 42 mM NaCl (NaCl), 10 mM citric acid (CA), 0.2 mM quinine, and 1.7 µM cycloheximide (bitter mix), 137 mM sucrose (sucrose), and 61 mM monosodium glutamate + 1 mM IMP (MSG). Because MSG contains 61 mM sodium, the MSG + IMP response was adjusted by estimating the amount of the response that could be due to sodium and subtracting it. The estimation was calculated by multiplying the response to 142 mM NaCl by 0.429, because 61 mM is 42.9% of 142 mM. This could be an overestimation of the salt component of the MSG response because it resulted in some negative values. If this resulted in a negative value, the response was reset to zero. To evaluate the correction factor for the umami stimulus, we examined 61 ROIs (from three mice) to 142 mM NaCl, 10 mM citric acid, 142 mM NH_4_Cl, 0.2 mM quinine and 1.7 µM cycloheximide (bitter mix),137 mM sucrose (sucrose), 142 mM NaCl, with 5 µM benzamil, and 61 mM monosodium glutamate + 1 mM IMP with 5 µM benzamil. In *Pou2f3*^−/−^ mice, we collected 90 ROIs for six stimuli (five basic taste stimuli and 142 mM NH_4_Cl) and 56 ROIs for five stimuli and two mixtures.

Since the absolute level of fluorescence can be influenced by the location of the cell in the ganglion or the level of GCaMP6s expression, the largest response for each neuron was set to 1.00, and other responses were reported relative to the maximal response. However, for low-responding neurons, this amplified nonsignificant responses. Those ROIs with no responses >0.1 Δ*F*/*F*0 to any stimuli after background subtraction, but before normalization, were excluded from analyses using normalized data principal component analysis (PCA), leaving 264 ROIs for five stimuli and 144 for seven stimuli.

Entropy (H) was calculated for each neuron and was averaged across neurons in the same cluster to yield the mean entropy per cluster. Entropy was calculated using normalized data such that the maximal response for each neuron is 1, and responses that were not significantly above baseline were set to 0. Entropy was calculated using responses to *n* = 6 taste stimuli; we did not use Na acetate in the calculation since it did not result in its own cluster. The same scaling constant (*K*) of 1.285097 for six stimuli was used to constrain the calculation to 0 < *H* ≤ 1 for all clusters. The proportion of a neuron’s response (pi) to each taste stimulus relative to the sum of all responses for that neuron was calculated. The log of pi was then multiplied by *pi* for the neuron's response to each stimulus. These values were summed across stimuli and then multiplied by *K*. For neurons that responded to only a single stimulus, *H* neared but did not equal 0. Higher values of *H* indicate greater breadth of tuning, but no neurons responded equally to all six stimuli.

To determine whether a mixture produced an additive response, the actual response was compared with the predicted additive response for each neuron. The predicted additive response was determined by adding the peak Δ*F*/*F*0 of the two components of the mixture, which is the predicted response if the components produced an additive response. To quantify deviations from linear summation, we calculated an additivity index (AI = *R*_mix_ / (*R*_A_ + *R*_B_)); values <1 indicate subadditive responses, values equal 1 indicate linear summation, and values >1 indicate superadditive responses. This logic has been explained previously (Hyman and Frank, 1980; Maier and Katz, 2013). Of the 135 ROIs analyzed from mixtures, 101 responded to either citric acid, sucrose, or the mixture, and responses from 34 neurons that did not respond to sucrose, citric acid, or the mixture were not included in this analysis. Of the 135 ROIs analyzed from mixtures, 84 responded to citric acid, NaCl, or the mixture, and 51 that did not respond to the mixture or its components were not included in this analysis. To examine lower concentrations of mixtures, an additional 62 ROIs (from two mice) were collected with the following stimuli: 10 mM citric acid, 60 mM NaCl, 60 mM sucrose, a mixture of 10 mM citric acid and 60 mM NaCl, and a mixture of 10 mM citric acid and 60 mM sucrose. Of these, 20 responded to either citric acid or sucrose and another 19 responded to both stimuli. Responses from 23 neurons that did not respond to the mixture or its components were not included in this analysis. Of these 62 ROIs, only four neurons responded to both citric acid and NaCl, so additivity ratios between these stimuli were not compared.

#### Statistical analysis

Hierarchical cluster analysis was performed in SPSS. We used Ward's method for linkage type, and Euclidean distance was the distance measure. The number of clusters was determined by dendrogram visualization combined with silhouette score. Based on the outcomes, individual neurons were assigned to groups and graphed in GraphPad Prism, where median responses for each stimulus were also calculated. We performed PCA using MATLAB and GraphPad Prism, which yielded similar results, so we report the outcomes from Prism. Spearman's correlations were used to analyze the relationships between variables, and correlation matrices were also produced in GraphPad Prism. Because the data were not normally distributed, differences between groups were tested using either a Mann–Whitney test (unpaired) or a Wilcoxon test (paired). Differences between groups in the normalized Δ*F*/*F* were compared for each stimulus using a Mann–Whitney test, with the number of comparisons controlled using Bonferroni's correction; actual *p* values were reported. Data are publicly available (https://data.mendeley.com/preview/xyhdd7ypsk?a=bede3722-1cf4-49cc-922a-7b2426f9745b).

## Results

### Taste-responsive neurons in the geniculate ganglion are narrowly or broadly tuned

Approximately half of the taste-responsive neurons in the geniculate ganglion are narrowly tuned (respond to a single stimulus), while the other half are broadly tuned (respond to more than one stimulus). The precise way in which taste information is represented by peripheral neurons has been debated for decades. However, numerous studies have demonstrated peripheral neurons innervating the tongue and/or palate have a wide range of tuning properties (Lundy and Contreras, 1999; Barretto et al., 2015; Wu et al., 2015). The exact distribution between broadly tuned and narrowly tuned neurons can vary depending on the concentrations of stimuli used in experiments (Wu et al., 2015).

To assess whether our findings align with these previous studies, we recorded calcium responses from 280 neurons in the geniculate ganglion, using stimulus concentrations previously defined as the half-maximal response (EC_50_) of the chorda tympani nerve to control for concentration (Wu et al., 2015; Fig. 1*A*). When examining the responses of these neurons to five different stimuli—each representing a basic taste category—we found that 47% responded to a single stimulus and 53% responded to more than one stimulus. These results are consistent with earlier studies of peripheral taste neuron function using calcium imaging (Barretto et al., 2015; Wu et al., 2015). Specifically, at medium concentration ranges, previous studies have found 51% of neurons to be broadly tuned and 49% to be narrowly tuned (Wu et al., 2015).

**Figure 1.**
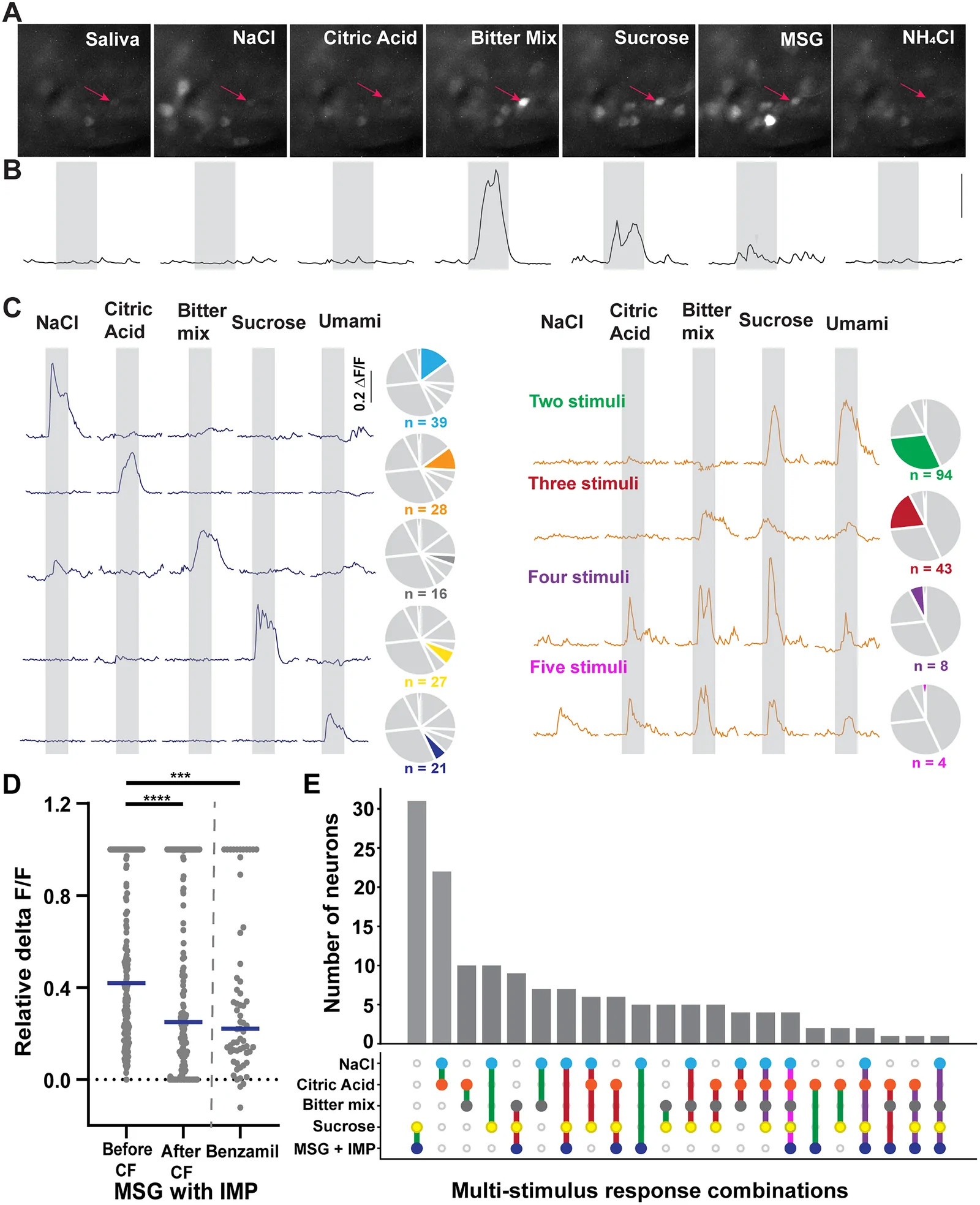
Gustatory neurons are either narrowly tuned or broadly tuned. ***A***, Representative images showing calcium responses in the geniculate ganglion evoked by taste stimulation of the tongue. The red arrow indicates a single neuron exhibiting changes in fluorescence (Δ*F*/*F*) across taste stimuli. ***B***, Time course of fluorescence change (Δ*F*/*F*) for the neuron indicated in (***A***). Gray bars denote the 10 s stimulus duration. ***C***, Example traces showing responses of narrowly tuned neurons (*n* = 131/280) that responded to a single stimulus (left) and broadly tuned neurons (*n* = 149/280) responding to multiple stimuli (right). Gray bars denote the 10 s stimulus duration. Pie charts on the left show the number of neurons responding to each individual stimulus among the narrowly tuned group. Pie charts on the right show the number of broadly tuned neurons responding to two (*n* = 94), three (*n* = 43), four (*n* = 8), or five stimuli (*n* = 4). The stimuli were 142 mM NaCl (NaCl), 10 mM citric acid, 0.2 mM quinine and 1.7 µM cycloheximide (bitter mix), 137 mM sucrose (sucrose), and 61 mM monosodium glutamate + 1 mM IMP (MSG). ***D***, Comparison of responses with 61 mM monosodium glutamate + 1 mM IMP before and after application of the correction factor (CF) and with 61 mM monosodium glutamate + 1 mM IMP in the presence of 5 µM benzamil. ***E***, The UpSet plot illustrates the number of broadly tuned neurons responding significantly to stimulus combinations. Colored dots indicate the stimuli to which neurons respond, and colored lines connect the stimuli, indicating that the neuron responds to two (green line), three (red line), four (purple line), or five stimuli (pink line). ****p ≤ 0.0001, ***p ≤ 0.001.

Despite strong overall agreement between our findings and the literature, one notable discrepancy emerged when comparing our data to a recent study reporting minimal umami-evoked activity in geniculate neurons (Fowler et al., 2022). In our experiments, we used MSG with IMP as the umami stimulus, whereas the previous study used MPG with IMP. This difference raises the possibility that our MSG responses might reflect sodium rather than glutamate or IMP. To account for the sodium present in MSG, we subtracted the estimated Na^+^-evoked component based on each neuron's response to NaCl—a correction that should, if anything, overestimate the sodium contribution, which significantly lowered responses to the umami stimulus (*W* = 6761, *p* < 0.0001; Fig. 1*D*). Even after applying this correction, umami remained a robust stimulus, with 27% of the neurons responding best to umami before the correction factor and 26% responding best to umami after the correction factor was applied (Fig. 1*D*; relative response = 1.0).

To further evaluate whether umami responses were driven by sodium, we recorded from 61 neurons using the same combination of 61 mM MSG and IMP but added 5 µM benzamil. Benzamil is an amiloride analog known to effectively block the ENaC-mediated sodium component of taste responses (Lundy and Contreras, 1997; Lyall et al., 2009; Breza and Contreras, 2012). Like the correction factor, this approach significantly lowered the response to umami stimuli (*U* = 4012, *p* < 0.0004; Fig. 1*D*), which is consistent with a sodium contribution to the response. There was no difference between responses to umami when the sodium contribution was subtracted using a correction factor and when sodium responses were inhibited by benzamil (*U* = 5691, *p* = 0.98). These data indicate that the correction factor from Wu et al. (2015) does a reasonably good job of reducing the impact of sodium on the response. The same proportion of neurons (7%) responded exclusively to umami in both conditions. The total proportion of neurons responding to the umami stimulus, using either the correction factor or benzamil, was 36% in both cases. In general, the correction factor appeared to reduce the impact of sodium on the umami response. However, in 18% of neurons, the correction factor reduced the umami response to below zero. Because peripheral neurons do not respond below baseline, we rectified these responses to 0. For these neurons, the correction factor overestimated the sodium contribution to the umami response. We elected to continue using the correction factor rather than adding benzamil to the MSG mixture because none of the 61 neurons tested with benzamil responded specifically to NaCl without benzamil; since NaCl is normally a robust stimulus, this finding suggests that benzamil remained on the tongue and altered responses to other stimuli in the array.

When examining data from broadly tuned neurons—those that respond to more than one stimulus—we observed that some combinations were much more common than others. The most common pair of stimuli eliciting responses was sucrose and umami (Fig. 1*E*). In contrast, only two neurons responded exclusively to citric acid and sucrose, and no neurons responded exclusively to bitter mix and umami. A total of 22 neurons responded to citric acid and bitter mix, but this group included both neurons that responded exclusively to this combination and those that responded to citric acid plus bitter mix along with one or more additional stimuli. In the subset of neurons that also had Na acetate as a stimulus, nearly as many neurons responded exclusively to NaCl and Na acetate as to sucrose and umami (Extended Data Fig. 1*E*-1). Of the 42 neurons that responded to NH_4_Cl, more than half (26) also responded to citric acid. When seven stimuli were tested, nine of the 24 neurons responding to the bitter mix also responded to NH_4_Cl. Together, these observations indicate that neurons respond to multiple stimuli in nonrandom, structured patterns. We speculate that the specific combinations of stimuli to which individual neurons respond may be important for how taste information is represented.

10.1523/ENEURO.0306-25.2026.f1-1 Figure 1-1 Stimulus–response combinations of broadly tuned neurons across seven stimuli. Colors indicate neurons are responsive to two stimuli (green), three stimuli (red), four stimuli (purple), five stimuli (pink), six stimuli (yellow), seven stimuli (gray). Download Figure 1-1, DOCX file.

### Cluster assignments are influenced by the number of taste stimuli included in the analysis

Peripheral gustatory neurons have been categorized into functional types using hierarchical cluster analysis (Lundy and Contreras, 1999; Breza et al., 2010; Wu et al., 2015). The number of stimuli used in these experiments is often limited. However, if the number of functional neuron types is an absolute feature of the neurons corresponding to genetically defined neuron types (Zhang et al., 2019), then the results would not depend on the number of stimuli used in the experiment.

We sought to determine whether hierarchical clustering consistently divides functional responses into five clusters corresponding to five genetically defined neuron types (Zhang et al., 2019) or whether the number of clusters is influenced by experimental design. Therefore, we performed hierarchical cluster analysis using responses to five taste stimuli (Fig. 2*A*) and compared the results to those obtained with seven taste stimuli. When five stimuli were used, neurons clustered into five clusters (Fig. 2*A*). However, when seven stimuli were included, the neurons formed six clusters (Fig. 2*B*), with ammonium chloride—but not sodium acetate—defining its own cluster. The NaCl cluster had the lowest entropy across the six clusters, indicating that these neurons are the least broadly tuned, even though this stimulus is well established to be mediated by multiple transduction mechanisms (Heck et al., 1984; Hill and Bour, 1985; Formaker and Hill, 1988; Chandrashekar et al., 2010; Oka et al., 2013; Lewandowski et al., 2016).

**Figure 2.**
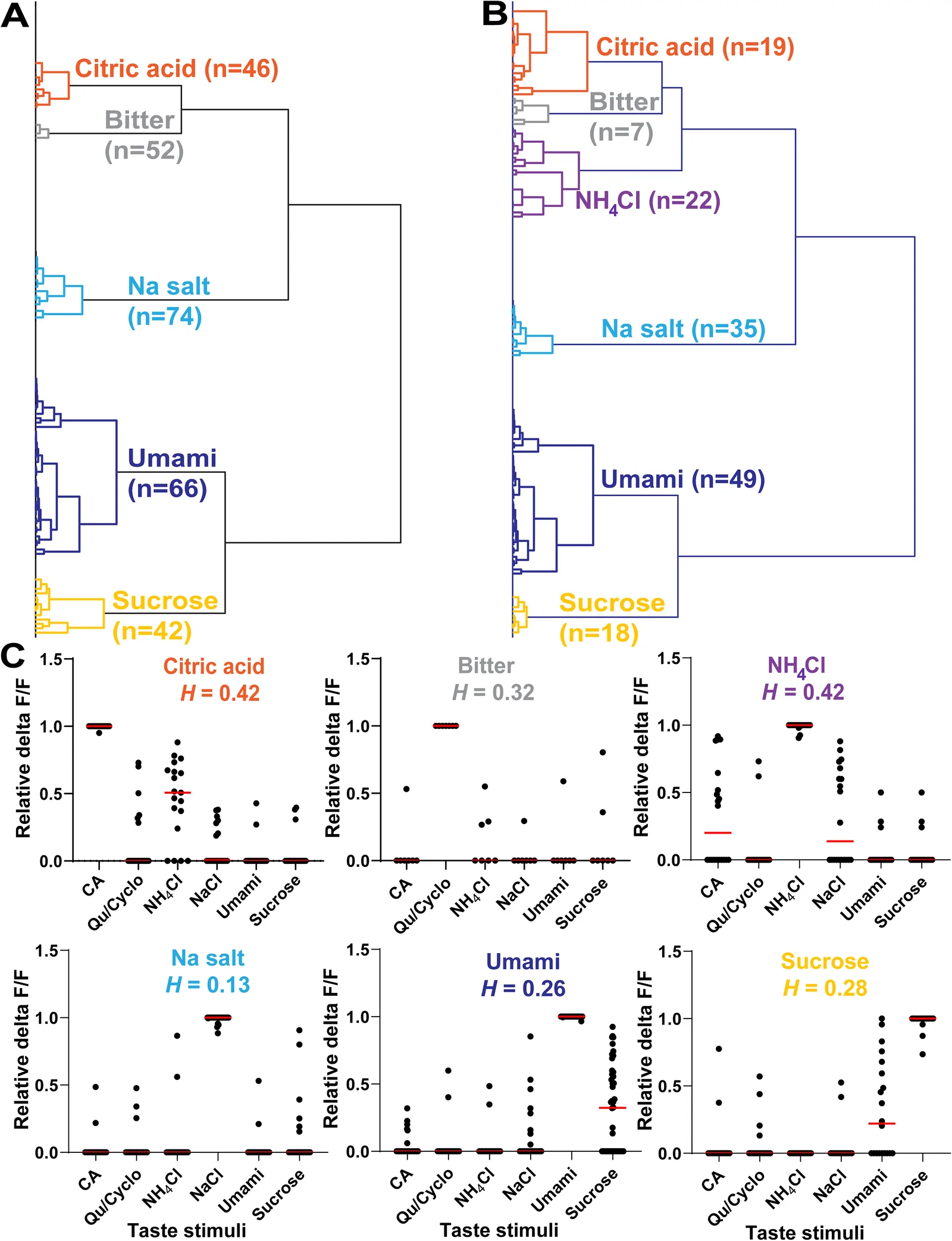
The number of functional neuron categories increases with the number of stimuli used. ***A***, Hierarchical cluster analysis based on neural responses to five taste stimuli (*n* = 280). ***B***, Hierarchical cluster analysis using responses to seven stimuli (*n* = 150). The addition of two stimuli results in one additional cluster, suggesting that the number of response categories increases with stimulus number, though not linearly. ***C***, Response profiles of neurons within each cluster defined in ***B***. Red lines indicate the median response for each stimulus. The five stimuli were 142 mM NaCl (NaCl), 10 mM citric acid, 0.2 mM quinine and 1.7 µM cycloheximide (bitter mix), 137 mM sucrose (sucrose), and 61 mM monosodium glutamate + 1 mM IMP (MSG), whereas the seven-stimulus analysis additionally included 142 mM NH_4_Cl and 142 mM sodium acetate. *H*-values denote the entropy calculated for each cluster.

This finding contradicts the idea that neurons are strictly divided into five clusters based on basic tastes (Zhang et al., 2019). Instead, our results suggest that the number of stimuli used in an experiment influences the number of neuronal groups identified by hierarchical cluster analysis. However, it is also the case that not all stimuli produce a unique cluster, since NaAc did not produce a cluster distinct from NaCl. Thus, clusters do not strictly represent the number of stimuli used in the experiment either. In addition, we observed that some clusters have median responses to a single stimulus, while others have median responses to two stimuli (Fig. 2*C*). Therefore, not every cluster corresponds exclusively to a single stimulus.

### Gustatory neurons function to classify taste stimuli into three categories

Both in the literature (Frank, 2000; Barretto et al., 2015; Wu et al., 2015) and in our data, broadly tuned neurons appear to respond to specific combinations of stimuli. To examine whether these stimulus combinations worked to group neurons together, we applied PCA, a statistical technique that identifies patterns in high-dimensional data by reducing its complexity. PCA transforms the original data into a set of new variables (principal components) that capture the most important variations in neural responses.

When a PCA was performed using seven stimuli, PC1 accounted for 40.47% of the variance and PC2 accounted for 21.25% of the variance. By plotting the loadings (Fig. 3*A*), it appears that taste responses naturally grouped into three distinct categories: caloric substances that animals prefer (sweet, umami), stimuli that mice typically avoid (ammonium chloride, citric acid, bitter), and sodium salts (NaCl, sodium acetate). When plotting the principal component values for individual neurons, both narrowly tuned and broadly tuned neurons contribute to the clusters (Fig. 3*B*). The clustering of neurons occurred because stimuli within the same category had positively correlated neural responses, while responses between categories were either uncorrelated or negatively correlated (Fig. 3*C*). Notably NaCl responses correlated with sodium acetate, but not with bitter mix or NH_4_Cl despite the findings that the amiloride insensitive portion of the NaCl response is transduced by cells sensitive to these compounds (Oka et al., 2013; Lewandowski et al., 2016). This pattern of three categories was consistent regardless of whether we used seven stimuli (Fig. 3*A–C*) or five stimuli (Fig. 3*D–F*).

**Figure 3.**
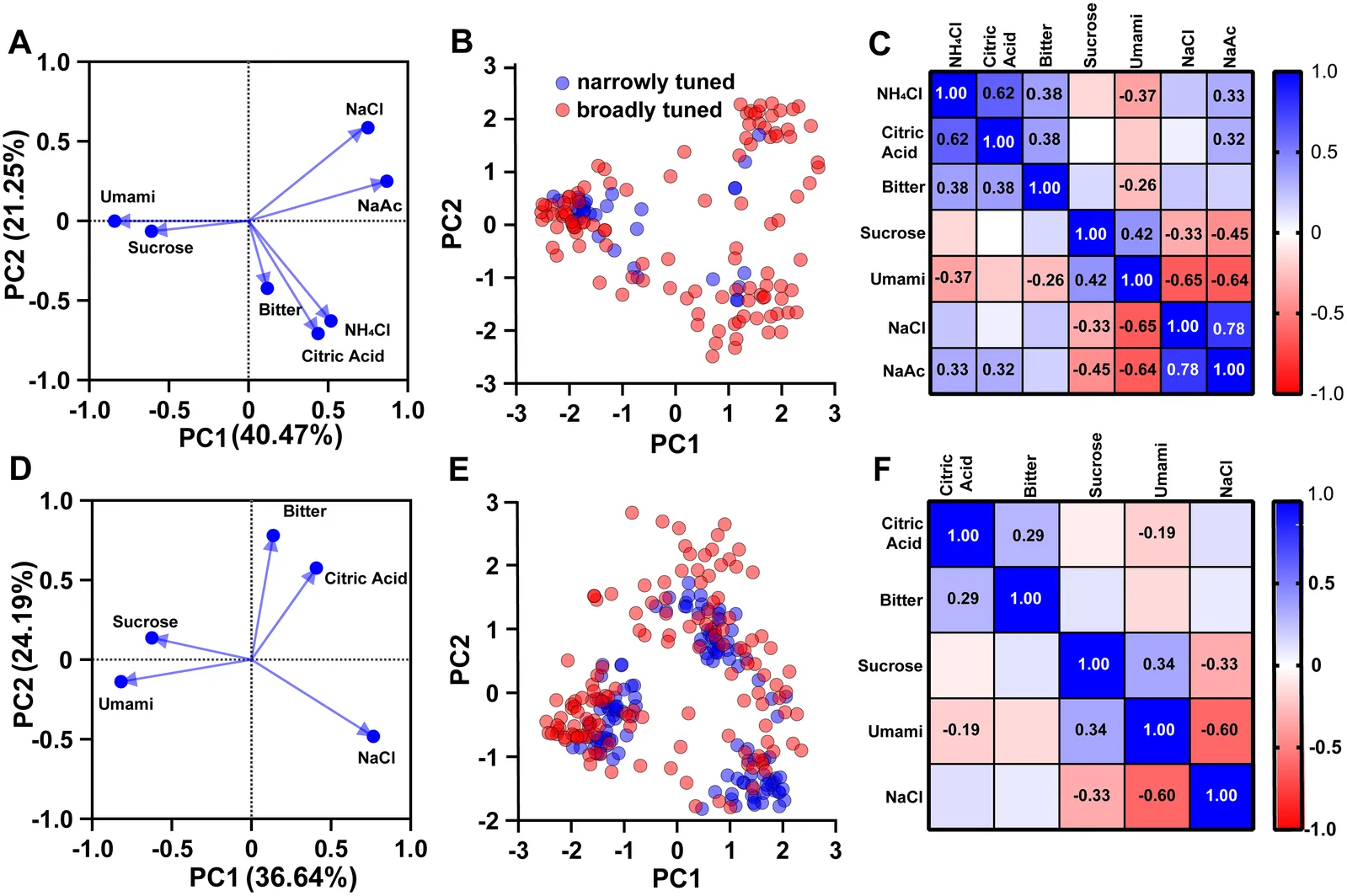
PCA reveals three major functional groups of neurons. ***A***, Correlation map of stimulus responses plotted in principal component space (F1 vs F2 eigenvalues) using responses to seven stimuli (*n* = 144). ***B***, Each neuron is plotted by its eigenvalues; blue dots represent neurons responding to a single stimulus, and red dots represent those responding to multiple stimuli. ***C***, Correlation within clusters is high, whereas correlation across clusters is low. ***D–F***, PCA results based on responses to five stimuli show clustering patterns similar to those observed in the seven-stimulus dataset (*n* = 264). Note: The proportion of narrowly tuned neurons depends on the number of stimuli tested.

Based on the degree of clustering, narrowly tuned neurons (Fig. 3*B*,*E*, blue) appeared to categorize gustatory stimuli more effectively than broadly tuned neurons (Fig. 3*B*,*E*, red). However, many broadly tuned neurons were grouped within one of the three stimulus categories, like the narrowly tuned neurons. Other broadly tuned neurons were scattered between the more well-defined categories, suggesting that they may respond to taste stimuli from more than one category.

### Gustatory neurons that are broadly tuned across stimulus categories have a nonadditive response to mixtures

We speculated that broadly tuned neurons with responses limited to within a stimulus category might function differently than those that respond to stimuli in different categories. To examine this idea more closely, we replotted Figure 3*E* and labeled neurons that responded only to bitter and sour stimuli or sweet and umami stimuli as green. This group of neurons comprised only 13% of the total neuron population but likely plays an important role in classifying neurons, as all were clustered within either the bitter-sour category or the sweet-umami category (Fig. 4*A*). The remaining 40% of the neurons were broadly tuned, responding to stimuli in more than one category. These neurons did not cluster into stimulus categories and instead were scattered throughout the stimulus space.

**Figure 4.**
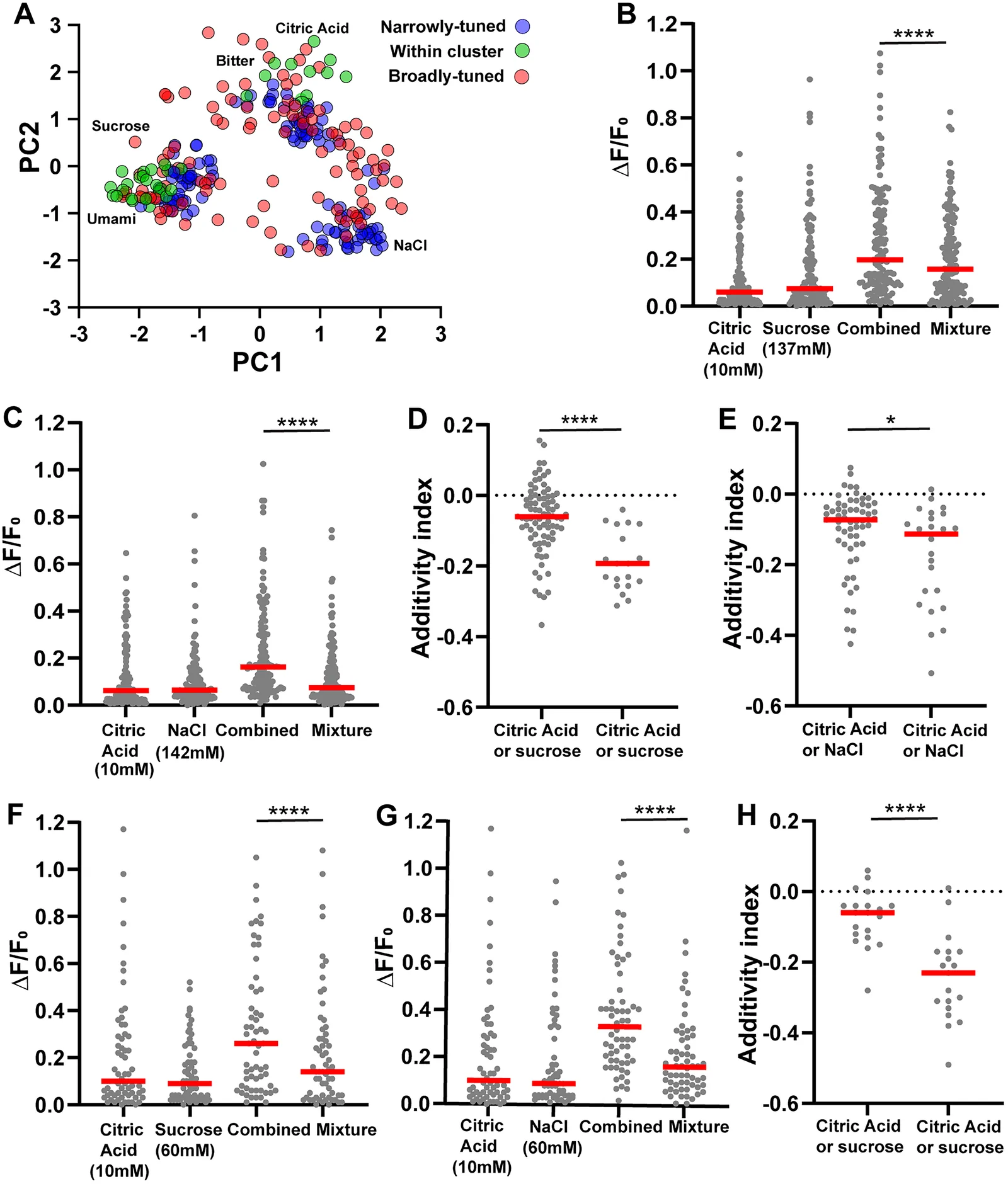
Broadly tuned neurons that respond to multiple stimulus categories contribute to mixture subadditivity. ***A***, Data from Figure 3*E* replotted to distinguish broadly tuned neurons responding to stimuli within a single category (green dots) from those responding to multiple categories (red dots), together with narrowly tuned neurons (blue dots). ***B***, ***C***, Mixture subadditivity is observed for two mixtures (*n* = 135) that combine stimuli from different categories: citric acid/sucrose mixtures (***B***) and citric acid/NaCl mixtures (***C***). ***D***, The additivity index calculated from the responses in panel ***B*** is plotted separately for neurons that respond to both citric acid and sucrose (*n* = 19) versus neurons that respond to sucrose or citric acid (*n* = 82), but not both. ***E***, The additivity index calculated from the responses in panel ***C*** is plotted separately for neurons that respond to both citric acid and NaCl (*n* = 24) versus those that respond to citric acid or NaCl (*n* = 60). ***F***, ***G***, Mixture subadditivity is still observed for citric acid/sucrose mixtures (***F***) and citric acid/NaCl mixtures (***G***) when the concentrations of sucrose and NaCl are reduced (*n* = 62). ***H***, The additivity index calculated from the responses in panel ***F*** is plotted separately for neurons that respond to both citric acid and sucrose (*n* = 19) versus neurons responding only to sucrose or citric acid (*n* = 20). This demonstrates reduced additivity of the mixture in neurons that respond to both stimuli. Red line, median. ****p ≤ 0.0001, *p ≤ 0.05.

We were surprised that as many as 40% of the neurons responded to stimuli from more than one category and wanted to explore the potential function of these neurons more thoroughly. We speculated that these neurons might respond differently than narrowly tuned neurons to mixtures of stimuli from multiple categories. It is well established in the field of taste that responses to mixtures are nonadditive (Bartoshuk, 1975; Formaker and Frank, 1996; Frank et al., 2003; Talavera et al., 2008; Breza and Contreras, 2012; Maier and Katz, 2013). What is interesting about subadditivity is that it varies in degree depending on both the combination of stimuli used in the mixture (Formaker and Frank, 1996; Formaker et al., 1997) and which neurons exhibit a nonadditive response (Breza and Contreras, 2012).

To verify that neurons demonstrated a nonadditive response with stimuli chosen across categories, we examined mixture responses for citric acid and sucrose (Fig. 4*B*) and citric acid with NaCl (Fig. 4*C*). For both mixtures, the response to the mixture was lower than the combined response (10 mM CA-137 mM sucrose *W* = −6751, *n* = 135, *p* < 0.0001; 10 mM CA-142 mM NaCl *W* = −8667, *n* = 135, *p* < 0.0001). In addition, there was considerable variation in the degree of subadditivity across neurons. This variation can be more easily observed by subtracting the predicted additive response from the measured mixture response (additivity index) for each neuron. We then compared the additivity index in neurons that responded across categories (to citric acid and sucrose or citric acid and NaCl) with neurons that only responded to one stimulus with the mixture (Fig. 4*D*,*E*). Neurons that responded to none of these stimuli were excluded. Neurons responding to both stimuli in the mixture exhibited substantially lower additivity than those responding to only one of the two stimuli (CA-sucrose, *U* = 270, *n* = 82, *n* = 19, *p* < 0.0001; CA-NaCl, *U* = 483.5, *n* = 60, *n* = 24, *p* < 0.0001; Fig. 4*D*,*E*).

One explanation for these mixture effects is a ceiling effect. The concentrations of the stimuli in each mixture were set at half of the maximum chorda tympani response. Although this leaves room for a higher response in the mixture, it is possible that many individual neurons were unable to respond at additive levels when both components were combined. To further explore these mixtures, we reduced the concentration of the stimulus that produced the highest response in each pair (sucrose and salt) to 60 mM. Mixture subadditivity remained robust at these lower concentrations (Fig. 4*F*,*G*; 10 mM CA-60 mM sucrose, *W* = 1794, *n* = 62, *p* < 0.0001; 10 mM CA-60 mM NaCl, *U* = 1006, *n* = 62, *p* < 0.0001). As seen at higher concentrations, when the additivity index for 60 mM sucrose and 10 mM citric acid were compared between neurons responding to one stimulus versus those responding to both, the additivity index was lower for neurons responding to both stimuli (10 mM CA-60 mM sucrose mixture, *U* = 45.5, *n* = 20, *n* = 19, *p* < 0.0001; Fig. 4*H*). However, only four neurons responded to both 60 mM NaCl and 10 mM citric acid, so the subadditive index was not compared for that mixture. Thus, one role of these broadly tuned neurons may be to represent mixtures differently from narrowly tuned neurons and broadly tuned neurons that respond only to stimuli within a single cluster.

### Type II cells are required for subadditivity of sweet/sour mixtures

We next set out to examine whether broadly tuned neurons receiving input from more than one category received converging input from Type II and Type III cells. To accomplish this goal, we took advantage of the requirement for the transcription factor *Pou2f3* for Type II taste cell differentiation. Mice lacking *Pou2f3* have a complete loss of Type II taste bud cells (Matsumoto et al., 2011; Larson et al., 2020; Fig. 5*A*). Reflecting this taste cell loss, all neurons in *Pou2f3^−/−^* had their highest response to either citric acid (*n* = 12) or ammonium chloride (*n* = 78; Fig. 5*B*). We found that functional responses to sucrose and umami stimuli are substantially reduced (sucrose, *U* = 4013, *n* = 144, 90, *p* < 0.0001; umami, *U* = 3596, *n* = 144, 90, *p* < 0.0001), while responses to citric acid and ammonium chloride are increased (CA, *U* = 3247, *n* = 144, 90, *p* < 0.0001; NH_4_Cl, *U* = 1082, *n* = 144, 90, *p* < 0.0001; Fig. 5*B*). Both acid and ammonium chloride have been shown to be transduced by *Otop1*, which is expressed by Type III cells (Teng et al., 2019; Liang et al., 2023). There was no difference between the two genotypes in responses to NaCl (*U* = 5771, *n* = 144, 90, *p* = 0.16) or bitter mix (*U* = 5687, *n* = 144, 90, *p* < 0.12). These two stimuli are likely transduced by a combination of Type II and Type III cells (Lewandowski et al., 2016; Dutta Banik et al., 2020).

**Figure 5.**
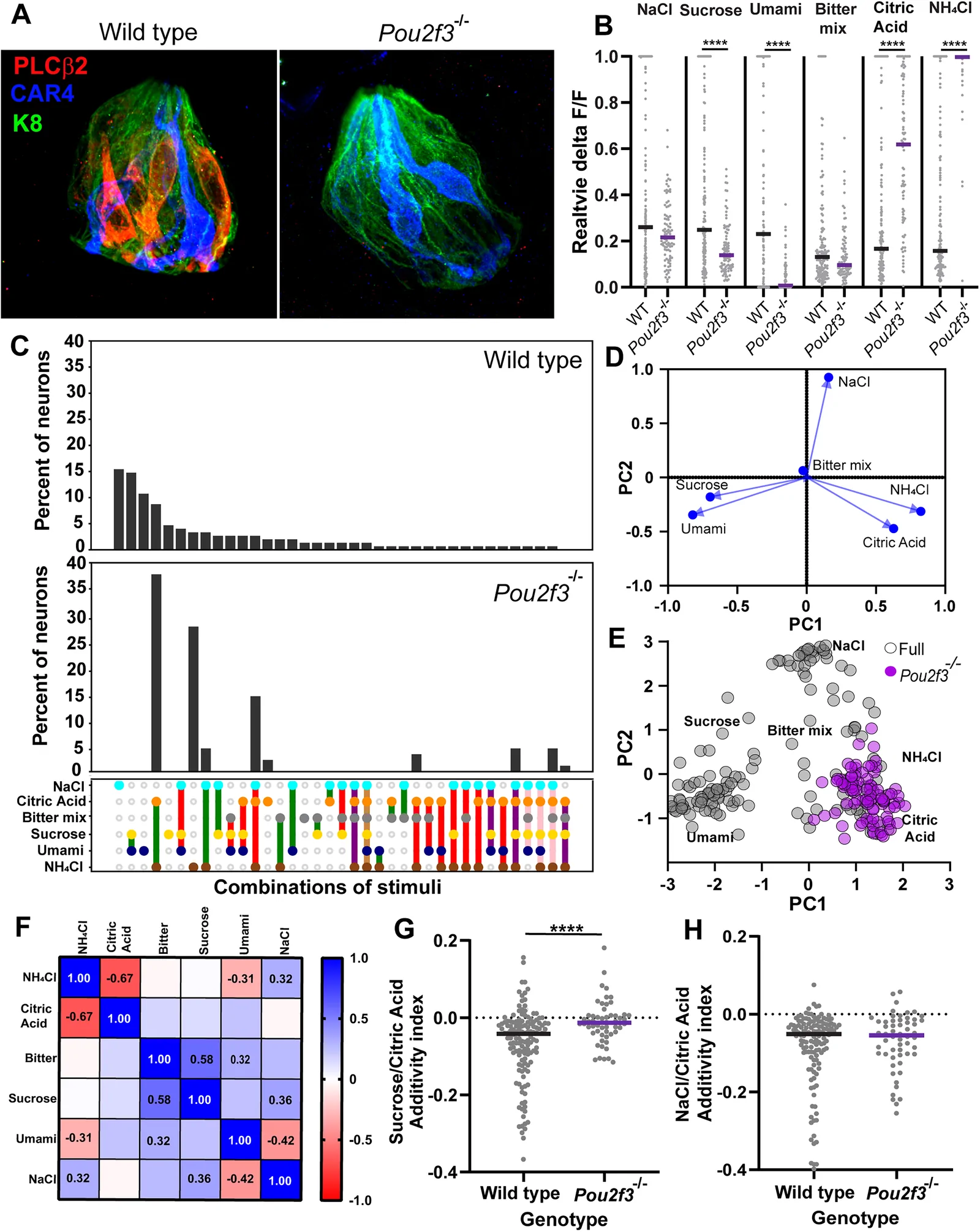
Convergent input from multiple taste cell types contributes to mixture subadditivity. ***A***, Image of taste buds showing that *Pou2f3*^−/−^ mice lack Type II taste cells expressing PLCβ2. ***B***, Loss of Type II cells in* Pou2f3*^−/−^ mice results in fewer neurons responding to sweet and umami stimuli and more neurons responding to citric acid and NH_4_Cl. Black and purple lines indicate medians (*Pou2f3*^−/−^, *n* = 90; WT, *n* = 144). ***C***, The UpSet plot illustrates the percentage of neurons responding significantly to single stimuli and stimulus combinations in wild-type mice (top) and *Pou2f3*^−/−^ mice (bottom). Colored dots indicate the stimuli to which neurons respond, and colored lines connecting the dots indicate neurons responding to two (green line), three (red line), four (purple line), or five stimuli (pink line). ***D***, Biplot showing stimulus categories when data from both wild-type and *Pou2f3*^−/−^ mice are used for PCA, indicating that Type II cells support the formation of an aversive stimulus category. However, when Type III cells are overrepresented, the bitter mix forms its own category. ***E***, Each neuron from the PCA in panel ***D*** is plotted by its eigenvalues, illustrating that, in the absence of Type II cells (purple), neurons fall into the NH_4_Cl–citric acid cluster. ***F***, Stimuli whose responses are correlated across neurons in wild-type mice (Fig. 3*C*; citric acid and NH_4_Cl, umami, and sucrose) do not have correlated responses in *Pou2f3*^−/−^ mice. ***G***, ***H***, Mixture subadditivity is reduced for citric acid–sucrose mixtures (***G***) but not citric acid–NaCl mixtures (***H***) in *Pou2f3*^−/−^ mice (*n* = 56 neurons) compared with wild-type mice (*n* = 135 neurons), suggesting that convergence of both Type II and Type III cells onto the same afferent fiber supports subadditivity across categories. Scale bar, 10 µm. ****p ≤ 0.0001.

While 40% of the neurons in wild-type mice were broadly tuned across categories, only 29% of neurons in *Pou2f3*^−/−^ mice were broadly tuned across categories. In addition, the combinations of different response possibilities were widely distributed in wild-type mice and more limited in *Pou2f3*^−/−^ mice (Fig. 5*C*). To examine the relationship between neuronal responses in wild-type and *Pou2f3*^−/−^ mice, we combined the datasets for PCA. By plotting the loadings, the results were similar to wild-type mice alone in that stimuli were grouped into appetitive and sodium categories. However, the bitter stimulus was separated from NH_4_Cl and citric acid, disrupting the aversive grouping (Fig. 5*D*). This was likely caused by the large number of citric acid– and ammonium chloride–responding neurons in *Pou2f3*^−/−^ mice (Fig. 5*C*). When plotting the principal component values, all neurons from *Pou2f3*^−/−^ mice were grouped in the cluster corresponding to citric acid and ammonium chloride (Fig. 5*E*, purple). This indicates that even for neurons that respond significantly to multiple stimuli in *Pou2f3*^−/−^ mice, citric acid/NH4Cl have the greatest influence on PC1 and PC2 values. Finally, because response profiles are skewed toward citric acid and NH_4_Cl, corelationships across neurons for different stimuli are altered in *Pou2f3*^−/−^ mice (compare Figs. 5*F*, 3*C*).

We next examined mixture subadditivity. If the neurons that show mixture subadditivity of citric acid/sucrose mixtures do so because they receive convergent information from Type II and Type III cells, then citric acid/sucrose mixtures should be closer to an additive response in *Pou2f3*^−/−^ mice compared with wild-type mice. We found that this was the case for citric acid/sucrose mixtures (*U* = 2318, *n* = 135, 56, *p* < 0.0001; Fig. 5*G*) but not for citric acid/NaCl mixtures (*U* = 3568, *n* = 135, 56, *p* = 0.54; Fig. 5*H*). This indicates that convergent information from Type II and Type III cells is required for subadditive responses to some mixtures but not others. For example, for salt and sour mixtures there is likely a direct impact of pH on transduction (Breza and Contreras, 2012; Formaker et al., 2012).

## Discussion

Peripheral taste neurons provide essential information to the brain about whether an ingested substance is palatable, aversive, toxic, or nutritive. The precise way this information is encoded has been debated for decades (Erickson, 1968; Hettinger and Frank, 1992; Alcantara et al., 1997; Contreras and Lundy, 2000; Smith et al., 2000; Spector and Travers, 2005; Yoshida et al., 2006; Tomchik et al., 2007; Frank et al., 2008; Yarmolinsky et al., 2009; Carleton et al., 2010; Barretto et al., 2015; Wu et al., 2015; Ohla et al., 2019). Early electrophysiological recordings from the taste nerves of several species (e.g., cats, dogs, rabbits, rats, and hamsters) failed to reveal a single neuron type dedicated to each of the basic taste qualities traditionally described in humans (Frank et al., 2008). Instead, gustatory neurons were divided into two major populations: narrowly tuned and broadly tuned neurons (Pfaffmann, 1941). Despite this foundational observation, peripheral taste coding has focused on whether neurons use a labeled-line or across-fiber pattern strategy, rather than on the potential role of neurons with differing tuning properties.

In the present study, we examined the calcium responses of geniculate ganglion neurons to determine how broadly tuned and narrowly tuned neurons contribute to peripheral taste representation. Hierarchical clustering grouped neurons based on their strongest response, but the number of resulting groups increased with the number of stimuli tested. This suggests that the peripheral taste system could potentially support the identification of many stimuli. In fact, these patterns, combined with spike timing and response latency, likely yield even more differences between individual stimuli (Di Lorenzo et al., 2003; Breza et al., 2010). However, PCA of our data indicates that peripheral neurons also categorize taste stimuli into three broad functional groups: innately aversive, caloric/innately appetitive, and sodium specific. These groups are both behaviorally relevant and consistent across many previous studies (Frank, 1973; Frank et al., 1988; Scott and Giza, 1990; Frank and Blizard, 1999; Lundy and Contreras, 1999; Contreras and Lundy, 2000; Frank, 2000; Smith et al., 2000; Frank et al., 2003; Frank et al., 2008; Barretto et al., 2015; Wu et al., 2015).

The same three-category organization was described previously (Frank et al., 1988; Frank, 2000), where neurons were grouped into response “types,” such as sucrose and sodium specialists, and sour-responsive generalist neurons. To some extent, this observed difference in tuning properties was determined by the stimuli used in the original experiment. Similar to our observations, sucrose-responsive neurons often respond to glutamate, and sodium-specific neurons respond to many different sodium salts, indicating potential for broader tuning (Frank, 2000; Frank et al., 2008). These categories correspond closely to our appetitive and sodium clusters. Sour-responsive neurons were originally labeled generalists because they also responded to ammonium chloride. This overlap is now understood to arise from shared transduction mechanisms, particularly the use of the same proton channel (e.g., Otop1) in both sour and ammonium chloride sensing (Teng et al., 2019; Liang et al., 2023). In addition, bitter stimuli often activate the same taste bud cells as sour stimuli, including both Type II and Type III cells (Barretto et al., 2015; Dutta Banik et al., 2020). Thus, this historical classification scheme tended to assign all broadly tuned neurons to a single “H” type (Frank, 2000; Frank et al., 2008).

Here we distinguish between two categories of broadly tuned neurons: those whose responses are confined to a single stimulus category and those that span multiple categories. We speculate that these populations serve distinct roles. Broadly tuned neurons with responses to stimuli limited to a single category likely function alongside narrowly tuned neurons to encode stimulus hedonic category. Together, these neurons comprise ∼60% of the geniculate population. This structure may allow animals to rapidly evaluate the hedonic value of a stimulus, supporting fast, adaptive behavioral responses.

This idea is supported by behavioral data: mice readily learn to discriminate sucrose from quinine, citric acid, and NaCl, suggesting that discriminations across categories are salient (Treesukosol et al., 2011). Mice can discriminate between quinine and citric acid, but only slightly above chance, suggesting that these two stimuli within the same category (aversive) are more perceptually similar. Mice can likely learn discriminations between many closely related stimuli based on differences in patterns (Erickson et al., 1994), temporal firing (Di Lorenzo et al., 2003), and response latency (Breza et al., 2010). However, we predict that discrimination between different stimuli within the same category (appetitive, aversive, and sodium) would be harder than distinguishing stimuli of different categories. Such findings would be consistent with the encoding strategy we propose and inconsistent with models that do not predict differences in discrimination difficulty based on palatability or stimulus category.

Behaviorally, there are two primary responses to taste stimuli: they are either consumed or rejected. Often those that are consumed address a bodily need (appetitive), and those that are rejected are not consumed even if doing so would address a bodily need (aversive). For example, mice avoid high concentrations of bitter and sour stimuli even when water deprived (Glendinning et al., 2002). This appetitive-versus-aversive distinction may represent an organizational principle that is conserved and enhanced centrally. For example, aversive oral temperatures converge with aversive tastes in the parabrachial nucleus (Li and Lemon, 2019; Li et al., 2022) and palatability is represented in cortex even though it can be changed by experience (Stone et al., 2022; Raymond et al., 2024). In the periphery, two categories for appetitive stimuli likely exist because these stimuli are consumed under different conditions. While sweet, umami, and sodium stimuli tend to be appetitive, sodium is often consumed in response to a need for sodium (Stricker and Wolf, 1969; Baumer-Harrison et al., 2020), while sweet and umami stimuli tend to be consumed in response to caloric need (Mook and Cseh, 1981). Together, these data suggest that the primary pattern of taste organization is based on hedonics or behavior and not perceived taste quality.

One difference between our findings and some recent literature in mice is that we observed robust responses to umami that correlated with sucrose, rather than sodium chloride. A recent study found no response to umami stimuli in the geniculate ganglion (Fowler et al., 2022). One possible explanation is that we used monosodium glutamate rather than monopotassium glutamate. Peripheral recordings in rodents have shown that responses to monosodium glutamate (MSG) can overlap with NaCl responses because MSG contains a sodium-dependent component that activates amiloride-sensitive salt pathways (Ninomiya and Funakoshi, 1989a,b). However, we attempted to mitigate this issue using a correction factor (Wu et al., 2015). We validated the correction factor by comparing it to MSG + IMP with benzamil which inhibits the sodium response and found robust responses in both instances. We do not currently have an explanation for why our umami responses were substantially more robust than those reported in the earlier study (Fowler et al., 2022). Our corrected umami responses did not correlate positively with sodium chloride; in fact, the correlation was negative. This suggests that our correction factor could have overestimated the contribution of sodium chloride to the response for some neurons. This could have occurred because MSG is a mixture of glutamate and sodium, and the responses are subadditive, potentially due to glutamate interfering with salt transduction.

While 60% of the neurons may function to define three categories based on hedonics, the remaining 40% of the neurons are broadly tuned across categories and likely serve a different function. We propose that these neurons encode contextual features such as stimulus intensity or mixture composition. Supporting this idea, these neurons are more likely to exhibit mixture subadditivity—a phenomenon in which the response to a mixture is less than the sum of responses to the individual components (Frank et al., 2003; Formaker et al., 2009). Notably, we found that mixture subadditivity is more pronounced in neurons that respond to both stimuli in the mixture, which primarily occurs in broadly tuned neurons. This suppressive interaction could prevent sensory overload and preserve perceptual contrast among categories in complex environments.

Mechanistically, mixture subadditivity may occur due to interactions within a taste bud cell (Talavera et al., 2008), cell–cell interactions in the taste bud (Roper, 2006; Dvoryanchikov et al., 2011; Huang and Wu, 2015), or interactions in the nerve fiber due to convergence (Miller, 1971). In addition, some mixture subadditivity could represent a ceiling effect, such that the combination of two responses exceeds either the neuron's capacity to respond or the ability to measure the response. The mechanism likely depends on the combination of the stimuli, stimulus concentrations, and whether convergence of two cell types onto the same neuron is required. Here we found that when Type II cells are removed, fewer neurons respond to both citric acid and sucrose. In addition, removal of Type II cells reduces the amount of mixture subadditivity for sucrose–citric acid mixtures. Therefore, it is likely that at least some of the mixture subadditivity occurring in sucrose–citric acid mixtures is due to convergence of Type II and Type III cells onto the same neurons. This type of connectivity has been shown to occur for a subset of neurons (Wilson et al., 2022).

In addition to mixture subadditivity, broadly tuned neurons may be particularly sensitive to lower intensities, where solutions can be detected but not identified (Bartoshuk, 1978). These neurons could respond to a wider range of stimuli as intensity increases (Ganchrow and Erickson, 1970; Wu et al., 2015). While many gustatory neurons increase their tuning properties with increasing intensity, not all do. Some neurons retain specific tuning properties even when high concentrations of taste stimuli are used (Wu et al., 2015). One possibility is that breadth of tuning increases with increasing intensity primarily within the 40% of broadly tuned neurons and that these neurons do not contribute much to stimulus identity or categorization.

Although convergence of multiple taste bud cell types onto one neuron may result in broadly tuned neurons, it is not the only possibility. Some neurons still respond to sucrose and bitter stimuli in *Pou2f3*^−/−^ mice despite the loss of Type II cells (Matsumoto et al., 2011; Larson et al., 2020). TRPM5 is expressed in Type II cells and has been shown to be important for sweet and bitter detection (Zhang et al., 2003). However, there is also considerable evidence that some residual transduction occurs for both sucrose (Damak et al., 2006; Ohkuri et al., 2009; Eddy et al., 2012) and bitter (Damak et al., 2006) in the absence of TRPM5. This residual response has been shown to be mediated by TRPM4, such that only the combined deletion of both TRPM4 and TRPM5 results in a complete loss of sensitivity to these stimuli (Dutta Banik et al., 2018). Furthermore, consistent with our findings, TRPM4 is expressed in Type III cells (Dutta Banik et al., 2018), and some Type III cells are broadly tuned (Tomchik et al., 2007) and have been shown to transduce these three stimuli (Dutta Banik et al., 2020; Dutta Banik and Medler, 2023). Here we show that in the absence of Type II cells, sucrose responses are significantly reduced while bitter responses are not. These data suggest that most sucrose transduction occurs in Type II cells; however, bitter transduction can occur in both cell types. Thus, TRPM4 in Type III cells may be more important for mediating bitter than sweet taste detection.

In most neural systems, neuron types are defined based on a combination of genetic markers, morphology, function, and developmental lineage (Abraira and Ginty, 2013; Le Pichon and Chesler, 2014; Zeng and Sanes, 2017). Such integrative classification has not yet been achieved in the peripheral taste system. Given the large number of broadly tuned neurons, it seems unlikely that each taste quality, as perceived by humans, corresponds to a genetically distinct peripheral neuron type. Instead, genetic identity may reflect connectivity or synaptic architecture. Taste-transducing cells in the epithelium—Type II and Type III cells—are defined not by the stimuli they detect but by their morphological, expression, and synaptic features (Murray and Murray, 1967; Delay et al., 1986; Murray, 1986; Finger, 2005; Wilson et al., 2017; Romanov et al., 2018; Wilson et al., 2022). These differences are likely mirrored in the gene expression profiles of the ganglion neurons that these taste bud cells connect with (Lee et al., 2017; Sukumaran et al., 2017; Hirose et al., 2020). Moreover, different geniculate neurons may contribute to distinct central circuits, further increasing the diversity of their transcriptional programs.

Although relationships between gene expression, morphology, and function are still unclear, we do have some hints. For example, it is intriguing that the gene *Penk* (encoding proenkephalin) is selectively expressed in neurons responsive to aversive stimuli, suggesting a molecular correlate for a functional category (Dvoryanchikov et al., 2017; Ohman et al., 2024). Whether similarly specific molecular markers exist for neurons encoding caloric or sodium-specific stimuli remains to be determined. Furthermore, we also know that peripheral gustatory neurons vary in morphology (Huang et al., 2021) and have speculated that broadly tuned neurons have axons that branch more elaborately in the periphery (Ohman and Krimm, 2021). These differences in branching characteristics are a permanent feature of the neuron (Whiddon et al., 2023) and likely underlie differences between neurons in functional convergence across taste buds (Miller, 1971). These findings indicate that broadly tuned neurons are either one or more separate populations. If so, genetic labels may also exist for subtypes within the broadly tuned population, reflecting differences in function or connectivity.

In summary, while peripheral taste neurons can distinguish a wide range of stimuli, they appear to serve a more fundamental role in categorizing stimuli into innately aversive, innately appetitive, and sodium-specific groups. This categorization is achieved by the combined actions of narrowly and broadly tuned neurons. A separate set of broadly tuned neurons may encode information about stimulus intensity and context, contributing to mixture subadditivity and helping to maintain perceptual clarity in complex taste environments. This framework moves beyond the labeled-line versus pattern-code dichotomy and is consistent with data from numerous studies in the field.

## Synthesis

Reviewing Editor: Arianna Maffei, Stony Brook University Decisions are customarily a result of the Reviewing Editor and the peer reviewers coming together and discussing their recommendations until a consensus is reached. When revisions are invited, a fact-based synthesis statement explaining their decision and outlining what is needed to prepare a revision will be listed below. The following reviewer(s) agreed to reveal their identity: Christian Lemon.

The reviewers agree that the data presented in the manuscript are a valuable contribution to the field. However, they have identified several issues with the data presentation, analysis and interpretation. The major issues are as follows:

1- there is a need for a better justification for the statistical analysis used and for the strategies for multivariate analysis. On this point, there are also imprecisions regarding how the Entropy value is presented. Line 195: Entropy logic is incorrect. Should be 0 < H <= 1. H can't be <= 0 unless the standard formula is modified. Formula and scaling constants for each N should also be reported. Line 665, how entropy is calculated for each cluster, as stated, is unclear.

2- The prevalence and characterization of broadly tuned neurons.

The reported fraction of "broadly tuned" GG neurons is strikingly high-apparently exceeding that of "low" range neurons in Wu et al. despite using concentrations near the paper's EC50 values. Please provide a full tabulation of response combinations for all broadly tuned neurons (i.e., which stimuli each of the 94 double, 43 triple, 8 quadruple neurons responded to). This would enable readers to evaluate the patterns of co-tuning.

3- Line 85: The concept of these three general neural categories is not new in taste and already demonstrated in multiple papers. The manuscript acknowledges this, but later in the document. Some attention to the prior literature should be given here, and how the present results fit in with what is already known.

4- HCA outcomes depend on linkage type and distance metric - these are not reported. Different dendrograms will result from different linkage approaches. Critically, how the number of groups was determined is also missing.

5- Results, first paragraph is counterintuitive. If the distribution of types depends on methods, it is not clear why it is stated that about half are broad or narrow.

6- Mixture suppression. The analysis framework is unclear. Please describe whether peak dF/F responses or the integral (area under curve) were used. Given GCaMP dynamics and stimulus strengths, ceiling effects could yield sub-additivity in mixtures. Also, displaying response traces for each individual cell in a supplemental figure would be useful.

7-Pou2f3 mutant data

a) Figure 5A indicates a complete loss of PLCβ2 TRCs in Pou2f3 mutants (consistent with Matsumoto et al., 2011), yet GG neurons still respond to sucrose and bitter mixtures. Prior work (e.g., Trpm5 KO - Zhang et al., 2003) argues against Type III cells mediating canonical bitter. This raises the possibility of non-taste-dependent or off-target responses in the imaging data. Please provide controls that could rule out trigeminal/somatosensory or mechanical artifacts (e.g., solution flow, temperature, etc).

b) Rather than plotting the pooled population (Fig. 5B), classify ROIs by their primary stimulus (as in Fig. 2C) and re-plot per class. This would make changes in tuning distributions and response magnitudes in Pou2f3 mutants more interpretable.

8- Figure 1B-C labels. Add x-axis (time) scales and clearly mark the stimulus epochs. The current traces suggest response-shape heterogeneity (e.g., sustained quinine/cycloheximide responses in bitter-only vs. triple-tuned neurons, with shorter responses in quadruple/quintuple cells; similar for sucrose). Also label cycloheximide in the figure.

9- Supplemental Table 1 is missing

10- Stimulus design and potential confounds for "broad tuning".

• NaCl at 146 mM. This is within a "high-salt" dose expected to activate both amiloride-sensitive and -insensitive pathways. According to Oka et al. (2013), high salt can activate ENaC TRCs alongside bitter and/or sour TRCs. Thus, the chosen "salt" stimulus is likely to produce multi-modal activation, complicating inferences about the origin of broad tuning.

• MSG at 61 mM. Equimolar glutamate and Na⁺ likely co-activate umami TRCs and ENaC TRCs. The methods describe a post hoc correction (multiplying by 0.429 and subtracting), but such transformations can introduce artifacts. Please justify and validate this correction, and consider repeating key experiments with monopotassium glutamate to avoid Na confounds

11- The analysis of mixture suppression is unclear. lease describe whether peak dF/F responses or the integral (area under curve) were used. Given GCaMP dynamics and stimulus strengths, ceiling effects could yield sub-additivity in mixtures. Also, displaying response traces for each individual cell in a supplemental figure would be useful.

12- It is unclear what panels 4D and 4E show. Why there are fewer points for the mixture responses?

13- Figure captions 5E-H lack clarity and detail

## Author Response

Response to the Reviewers:

We would like to thank the reviewers for their valuable comments and feel the manuscript has been greatly improved by addressing their concerns/questions.

The major issues are as follows:

1- there is a need for a better justification for the statistical analysis used and for the strategies for multivariate analysis. On this point, there are also imprecisions regarding how the Entropy value is presented. Line 195: Entropy logic is incorrect. Should be 0 < H <= 1. H can't be <= 0 unless the standard formula is modified. Formula and scaling constants for each N should also be reported. Line 665, how entropy is calculated for each cluster, as stated, is unclear.

We tried to better justify the analyses. We have corrected errors and elaborated on how entropy was calculated in the methods section. We used 6 stimuli and the same scaling constant of 1.285097 for all clusters.

2- The prevalence and characterization of broadly tuned neurons.

The reported fraction of "broadly tuned" GG neurons is strikingly high-apparently exceeding that of "low" range neurons in Wu et al. despite using concentrations near the paper's EC50 values. Please provide a full tabulation of response combinations for all broadly tuned neurons (i.e., which stimuli each of the 94 double, 43 triple, 8 quadruple neurons responded to). This would enable readers to evaluate the patterns of co-tuning.

The EC50 values reported by Wu et al. were 136 mM sucrose, 142 mM NaCl, 61 mM MSG (plus 1 mM IMP); 7 mM citric acid, 0.2 mM quinine HCl, 1.7 μM cycloheximide plus .2mM quinine HC1, which is what we used although we used 10mM citric acid instead of 7mM. These values are very close to the middle values of Wu et al. The low values in Wu et al. were lower 100 mM sucrose; 60 mM NaCl; 3 mM citric acid; mixture of 0.6 μM cycloheximide with 0 or 0.1 mM quinine; 60 mM monosodium glutamate with 1 mM IMP. So, the most comparable data to our data are with the middle values of Wu et al. They found that 51% of the neurons were broadly tuned, while we found that 53% were broadly tuned. We thought this was very close.

A full tabulation of over 50 response combinations would be tedious to read. I tried making Venn diagrams for 7 stimuli but could not fit in every combination. The raw data are now provided. So, instead of providing a value for every combination we tried to provide a new paragraph with a few combinations that were noticeably more common than others. I think this answers the question the reviewer has. In addition, this is why we did the PCA, so explaining this better justifies the PCA while maintaining readability of the manuscript.

3- Line 85: The concept of these three general neural categories is not new in taste and already demonstrated in multiple papers. The manuscript acknowledges this, but later in the document. Some attention to the prior literature should be given here, and how the present results fit in with what is already known.

Change made as suggested.

4- HCA outcomes depend on linkage type and distance metric - these are not reported. Different dendrograms will result from different linkage approaches. Critically, how the number of groups was determined is also missing.

This information has been added to the methods section.

5- Results, first paragraph is counterintuitive. If the distribution of types depends on methods, it is not clear why it is stated that about half are broad or narrow.

This paragraph has been re-worded.

6- Mixture suppression. The analysis framework is unclear. Please describe whether peak dF/F responses or the integral (area under curve) were used.

We used peak dF/F responses. This information has been added to the methods.

Given GCaMP dynamics and stimulus strengths, ceiling effects could yield sub-additivity in mixtures.

This could be one potential cause of mixture suppression in most studies, since all neurons also have a maximum response rate, regardless of the measure. This possibility has been added to the discussion. In addition, we added data to the results with lower concentrations of stimuli to further explore this issue. However, we do not claim to rule out a ceiling effect, since I do not think that is possible. Lastly, I would point out that even if mixture suppression is due to a ceiling effect, this effect is still present in the neural representation that the brain receives.

Also, displaying response traces for each individual cell in a supplemental figure would be useful.

Unless I am misunderstanding reviewers are requesting a Figure where each of 21 responses for 551 neurons (11571 responses) are plotted. I am pretty sure that I cannot fit that on a single Figure, and I think it should require more justification than "it would be useful". For all data we analyzed a peak response, and we did not try to make any claims about adaptation properties or other measures best achieved with electrophysiology.

7-Pou2f3 mutant data a) Figure 5A indicates a complete loss of PLCβ2 TRCs in Pou2f3 mutants (consistent with Matsumoto et al., 2011), yet GG neurons still respond to sucrose and bitter mixtures. Prior work (e.g., Trpm5 KO - Zhang et al., 2003) argues against Type III cells mediating canonical bitter. This raises the possibility of non-taste-dependent or off-target responses in the imaging data. Please provide controls that could rule out trigeminal/somatosensory or mechanical artifacts (e.g., solution flow, temperature, etc).

We have a control for the Skn1a mice, which are wild type mice and responses are best considered in terms of the comparison between the two. We show considerable loss of sweet responses, but not a significant reduction in bitter.

We have added the following information concerning stimulation procedures to the methods: Taste stimuli were delivered using a programable perfusion device with 8 reservoirs, one of which held artificial saliva and the others held taste stimuli. All solutions were mixed and delivered at room temperature of 26wC. Artificial saliva in one reservoir was perfused constantly over the tongue surface. For 10 s of every minute, the solenoid valve for artificial saliva closes and one for a taste stimulus opens. Once this occurs a taste stimulus was infused over the tongue through the same infusion pump, such that flow rate is held constant.

We have added the following paragraph to the discussion: Although responses to sucrose and sucrose/citric acid mixture were substantially reduced in Pou2f3--/- mice, some neurons still respond to sucrose and bitter stimuli despite the loss of Type II cells (Matsumoto et al., 2011; Larson et al., 2020). TRPM5 is expressed in Type II cells and has been shown to be important for sweet and bitter detection (Zhang et al., 2003). However, there is also considerable evidence that some residual transduction occurs for both sucrose (Damak et al., 2006; Ohkuri et al., 2009; Eddy et al., 2012) and bitter (Damak et al., 2006) in the absence of TRPM5. This residual response has been shown to be mediated by TRPM4, such that only the combined deletion of both TRPM4 and TRPM5 result in a complete loss of sensitivity to these stimuli (Dutta Banik et al., 2018). Furthermore, consistent with our findings TRPM4 is expressed in Type III cells (Dutta Banik et al., 2018) and some Type III cells are broadly-tuned (Tomchik et al., 2007) and have been show to transduce these three stimuli (Dutta Banik et al., 2020; Dutta Banik and Medler, 2023). Here we show that in the absence of Type II cells sucrose responses are significantly reduced while bitter responses are not. These data suggest that most sucrose transduction occurs in Type II cells; however, bitter transduction can occur in both cell types. Thus, TRPM4 in Type III cells may be more important for meditating bitter than sweet taste detection. b) Rather than plotting the pooled population (Fig. 5B), classify ROIs by their primary stimulus (as in Fig. 2C) and re-plot per class. This would make changes in tuning distributions and response magnitudes in Pou2f3 mutants more interpretable.

We are not sure of your definition of "class". We plotted clusters in Fig 2C. However, based on the data, clusters will not occur in the Pou2f3 mutants, just like you don't get three groups using PCA. We added the following statement for clarity: Reflecting this taste cell loss, all neurons in Pou2f3-/- had their highest response to either citric acid (n=12) or ammonium chloride (n=78; Figure 5B)." Note none of the responses for neurons from Pou2f3-/- reach a relative response of 1 for NaCl, sucrose, umami, or bitter mix.

8- Figure 1B-C labels. Add x-axis (time) scales and clearly mark the stimulus epochs. The current traces suggest response-shape heterogeneity (e.g., sustained quinine/cycloheximide responses in bitter-only vs. triple-tuned neurons, with shorter responses in quadruple/quintuple cells; similar for sucrose). Also label cycloheximide in the figure.

Gray bars have been added to indicate the duration of the stimulus which was 10 seconds and so this adds a time scale. The traces represent an example of a single neurons, and so it is difficult to know whether that applies generally. In general, peak response and area under the curve are well corelated. However, we have not measured rate of response decrease relative to rinse application or rate of response onset following stimulus application. These could be useful additional measures. However, they are beyond our current scope, since we would not be able to complete the programming required and reanalysis in the 3 month revision period. However, we plan to do this in the future.

9- Supplemental Table 1 is missing We were referring to a data set, not a table, it has been uploaded for publication here. https://data.mendeley.com/datasets/nry4p7yy8k/1 10- Stimulus design and potential confounds for "broad tuning".

NaCl at 146 mM. This is within a "high-salt" dose expected to activate both amiloride-sensitive and -insensitive pathways. According to Oka et al. (2013), high salt can activate ENaC TRCs alongside bitter and/or sour TRCs. Thus, the chosen "salt" stimulus is likely to produce multi-modal activation, complicating inferences about the origin of broad tuning.

The concentration of 146mM NaCl is half maximum of the chorda tympani response, so it is neither low or high, but medium. There are at least two transduction mechanisms for NaCl. However, having two transduction mechanisms for a single stimulus does not confound the breadth of tuning for the neuron, A neuron either responds to one stimulus, or it does not. The information the brain receives is whether or not the neuron responded or didn't to the stimulus, regardless of the number of transduction mechanisms. Two transduction mechanisms for a single stimulus could potentially explain why a neuron is more broadly tuned. Interestingly, it does not in this case. Neurons in the NaCl cluster had the lowest entropy of any cluster, indicating they were the most specific. In addition, responses across all neurons to NaCl (sodium salt) and Na acetate (sodium salt) were significantly co-related while responses between NaCl (sodium salt) and NH4Cl (non-sodium salt) were not. This indicates to me that sodium transduction (amiloride sensitive) mechanisms likely play a larger role in the responses to 146mM sodium chloride than non-sodium mechanisms (amiloride insensitive). We added this information the results section.

MSG at 61 mM. Equimolar glutamate and Na⁺ likely co-activate umami TRCs and ENaC TRCs. The methods describe a post hoc correction (multiplying by 0.429 and subtracting), but such transformations can introduce artifacts. Please justify and validate this correction, and consider repeating key experiments with monopotassium glutamate to avoid Na confounds Yes, unfortunately this is a mixed stimulus. The underlying assumption of reducing the Umami response by the predicted amount due to sodium based on the sodium chloride response assumes linear responses for NaCl. That is the responses to 61mM sodium may not be 43% of the responses to 142mM NaCl, even though the concentration of NaCl is 43% lower. In addition, this calculation does not account for the non-sodium component of the sodium chloride response. As a result, it likely overestimates the amount of the response due to sodium, which is why we had to rectify some of the responses back to zero. However, it is unclear why using monopotassium glutamate would resolve the issue, since the taste system responds to potassium, we would then need to estimate the response due to potassium and subtract it. Unfortunately, we lacked potassium salts as a stimulus so had no ability to estimate this. We attempted to address this issue with an additional experiment in which benzamil is added to MSG and IMP to block the sodium response. These data have been added to the results.

11- The analysis of mixture suppression is unclear. please describe whether peak dF/F responses or the integral (area under curve) were used. Given GCaMP dynamics and stimulus strengths, ceiling effects could yield sub-additivity in mixtures. Also, displaying response traces for each individual cell in a supplemental figure would be useful.

These issues were all addressed in earlier comments, specifically #6.

12- It is unclear what panels 4D and 4E show. Why are there fewer points for the mixture responses? An explanation of mixture suppression calculations was added to the methods and the Figure legend for 4D and 4E were revised for clarity. Panels D and E compare the neurons that respond to both components of the mixture to the ones that do not; fewer neurons respond to both components.

13- Figure captions 5E-H lack clarity and detail These captions have been rewritten to provide more information.

Damak S, Rong M, Yasumatsu K, Kokrashvili Z, Perez CA, Shigemura N, Yoshida R, Mosinger B, Jr., Glendinning JI, Ninomiya Y, Margolskee RF (2006) Trpm5 null mice respond to bitter, sweet, and umami compounds. Chem Senses 31:253-264.

Dutta Banik D, Medler KF (2023) Defining the role of TRPM4 in broadly responsive taste receptor cells. Front Cell Neurosci 17:1148995.

Dutta Banik D, Martin LE, Freichel M, Torregrossa AM, Medler KF (2018) TRPM4 and TRPM5 are both required for normal signaling in taste receptor cells. Proc Natl Acad Sci U S A 115:E772-E781.

Dutta Banik D, Benfey ED, Martin LE, Kay KE, Loney GC, Nelson AR, Ahart ZC, Kemp BT, Kemp BR, Torregrossa AM, Medler KF (2020) A subset of broadly responsive Type III taste cells contribute to the detection of bitter, sweet and umami stimuli. PLoS Genet 16:e1008925.

Eddy MC, Eschle BK, Peterson D, Lauras N, Margolskee RF, Delay ER (2012) A conditioned aversion study of sucrose and SC45647 taste in TRPM5 knockout mice. Chem Senses 37:391-401.

Larson ED, Vandenbeuch A, Anderson CB, Kinnamon SC (2020) Function, Innervation, and Neurotransmitter Signaling in Mice Lacking Type-II Taste Cells. eNeuro 7.

Matsumoto I, Ohmoto M, Narukawa M, Yoshihara Y, Abe K (2011) Skn-1a (Pou2f3) specifies taste receptor cell lineage. Nat Neurosci 14:685-687.

Ohkuri T, Yasumatsu K, Horio N, Jyotaki M, Margolskee RF, Ninomiya Y (2009) Multiple sweet receptors and transduction pathways revealed in knockout mice by temperature dependence and gurmarin sensitivity. Am J Physiol Regul Integr Comp Physiol 296:R960-971.

Tomchik SM, Berg S, Kim JW, Chaudhari N, Roper SD (2007) Breadth of tuning and taste coding in mammalian taste buds. J Neurosci 27:10840-10848.

Zhang Y, Hoon MA, Chandrashekar J, Mueller KL, Cook B, Wu D, Zuker CS, Ryba NJ (2003) Coding of sweet, bitter, and umami tastes: different receptor cells sharing similar signaling pathways. Cell 112:293-301.
