# Supplementary material for: Narrowly and Broadly Tuned Peripheral Gustatory Neurons Cooperate to Encode Three Behaviorally Relevant Stimulus Categories
Source: eNeuro. 2026 Aug 19;13(8):ENEURO.0306-25.2026. doi: 10.1523/ENEURO.0306-25.2026 (PMC13631774; doi:10.1523/ENEURO.0306-25.2026)
Supplement: Supplementary file 1 [file eneuro-13-ENEURO.0306-25.2026-s002.docx]

Extended data, Figure 1-1: Stimulus–response combinations of broadly tuned neurons across seven stimuli. Colors indicate neurons are responsive to two stimuli (green), three stimuli (red), four stimuli (purple), five stimuli (pink), six stimuli (yellow), seven stimuli (gray).

| # of Neurons | NH_4_Cl | Citric  Acid | Bitter | Sucrose | Umami | NaCl | Na  Acetate |
| --- | --- | --- | --- | --- | --- | --- | --- |
| 22 |  |  |  |  |  |  |  |
| 19 |  |  |  |  |  |  |  |
| 11 |  |  |  |  |  |  |  |
| 5 |  |  |  |  |  |  |  |
| 4 |  |  |  |  |  |  |  |
| 4 |  |  |  |  |  |  |  |
| 4 |  |  |  |  |  |  |  |
| 4 |  |  |  |  |  |  |  |
| 3 |  |  |  |  |  |  |  |
| 3 |  |  |  |  |  |  |  |
| 3 |  |  |  |  |  |  |  |
| 3 |  |  |  |  |  |  |  |
| 2 |  |  |  |  |  |  |  |
| 2 |  |  |  |  |  |  |  |
| 2 |  |  |  |  |  |  |  |
| 2 |  |  |  |  |  |  |  |
| 2 |  |  |  |  |  |  |  |
| 2 |  |  |  |  |  |  |  |
| 1 |  |  |  |  |  |  |  |
| 1 |  |  |  |  |  |  |  |
| 1 |  |  |  |  |  |  |  |
| 1 |  |  |  |  |  |  |  |
| 1 |  |  |  |  |  |  |  |
| 1 |  |  |  |  |  |  |  |
| 1 |  |  |  |  |  |  |  |
| 1 |  |  |  |  |  |  |  |
| 1 |  |  |  |  |  |  |  |
| 1 |  |  |  |  |  |  |  |
| 1 |  |  |  |  |  |  |  |
| 1 |  |  |  |  |  |  |  |
| 1 |  |  |  |  |  |  |  |
| 1 |  |  |  |  |  |  |  |
| 1 |  |  |  |  |  |  |  |
| 1 |  |  |  |  |  |  |  |
| 1 |  |  |  |  |  |  |  |
